# Effects of androgen receptor signaling inhibitors on absorbed doses in mCRPC patients undergoing [^177^Lu]Lu-PSMA-617 or [^177^Lu]Lu-PSMA-I&T therapy: dosimetry results

**DOI:** 10.1007/s00259-026-07967-3

**Published:** 2026-06-11

**Authors:** Josef Zahner, Astrid Delker, Zachary Ells, Ana Antic Nikolic, Adrien Holzgreve, Sophie C. Siegmund, Maximilian Tiling, Mathias J. Zacherl, Elena Berg, Can D. Aydogdu, Alexander Dierks, Marcus Unterrainer, Guido Böning, Christian Stief, Jeremie Calais, Constantin Lapa, Rudolf A. Werner, Jozefina Casuscelli, Lena M. Unterrainer

**Affiliations:** 1 Department of Nuclear Medicine, LMU University Hospital, LMU Munich, Marchioninistrasse 15, 81377 Munich, Germany; 2 Ahmanson Translational Theranostics Division, Department of Molecular and Medical Pharmacology, David Geffen School of Medicine, UCLA, Los Angeles, USA; 3 Department of Nuclear Medicine, University Hospital Augsburg, Augsburg, Germany; 4 Department of Urology, LMU University Hospital, LMU Munich, Munich, Germany; 5 Die Radiologie, Munich, Germany

**Keywords:** MHSPC, ARSi, Dosimetry, Absorbed dose

## Abstract

**Background:**

Androgen receptor signaling inhibitors (ARSi) have significantly improved clinical outcomes in men with prostate cancer (PCa). Preliminary data suggest the potential for ARSi to enhance PSMA expression in patients with mCRPC. In this analysis, we present preliminary dosimetry results from mCRPC patients treated with either [^177^Lu]Lu-PSMA-617 or [^177^Lu]Lu-PSMA-I&T radioligand therapy (RLT) in combination with ARSi compared to a Control Group of patients receiving [^177^Lu]Lu-PSMA-617 or [^177^Lu]Lu-PSMA-I&T monotherapy.

**Methods:**

This retrospective analysis was performed at a single center, including 50 patients diagnosed with mCRPC who underwent their first cycle of [^177^Lu]Lu-PSMA-617 (n = 37) or [^177^Lu]Lu-PSMA-I&T (n = 13) RLT ± concomitant ARSi treatment (abiraterone or enzalutamide). Patients were divided into a Combination Group (RLT + ARSi, *n* = 25) and a Control Group (RLT only, *n* = 25). Quantitative SPECT imaging was performed at 3 time points: 24 h (+ CT), 48 h, and 72 h post-administration of the initial cycle of RLT. Tumor lesions were segmented using the 24 h SPECT images, while organs-at-risk (kidney, spleen, liver) were delineated on the corresponding CT. Absorbed doses were calculated by applying a mono-exponential curve fit, incorporating local density scaling. Dosimetry results were evaluated separately for each radioligand.

**Results:**

Overall, 148 tumor lesions were included in this analysis (median 3 lesions per patient). The median tumor absorbed dose was comparable between the Combination Group and the Control Group: [^177^Lu]Lu-PSMA-617 (2.1 ± 4.0 Gy/GBq vs 1.5 ± 1.7 Gy/GBq, *p =* 0.24) and [^177^Lu]Lu-PSMA-I&T (1.2 ± 1.2 Gy/GBq vs 1.2 ± 3.5 Gy/GBq, *p =* 0.21). Furthermore, the median absorbed dose in organs-at-risk was comparable between the two groups in [^177^Lu]Lu-PSMA-617: Kidneys: 0.28 ± 0.11 Gy/GBq vs 0.27 ± 0.13 Gy/GBq, *p =* 0.90; Spleen: 0.05 ± 0.05 Gy/GBq vs 0.06 ± 0.05 Gy/GBq, *p =* 0.75; Liver: 0.08 ± 0.03 Gy/GBq vs 0.07 ± 0.04 Gy/GBq, *p =* 0.75). A statistically significant difference was noted in kidneys and liver for [^177^Lu]Lu-PSMA-I&T: Kidneys: 0.21 ± 0.10 Gy/GBq vs 0.30 ± 0.08 Gy/GBq, *p =* 0.01; Spleen: 0.02 ± 0.02 Gy/GBq vs 0.04 ± 0.03 Gy/GBq, *p =* 0.20; Liver: 0.02 ± 0.01 Gy/GBq vs 0.04 ± 0.01 Gy/GBq, *p =* 0.01.

**Conclusion:**

In this pilot cohort, concomitant ARSi therapy did not significantly enhance tumor absorbed doses in patients receiving [^177^Lu]Lu-PSMA-617 or [^177^Lu]Lu-PSMA-I&T RLT. Further prospective studies with larger patient numbers are needed to evaluate the impact of concomitant ARSi treatment on the absorbed doses in mCRPC tumor lesions.

**Supplementary Information:**

The online version contains supplementary material available at https://doi.org/10.1007/s00259-026-07967-3.

## Introduction

According to the EAU-EANM-ESTRO-ESUR-ISUP-SIOG Guidelines on prostate cancer, patients with metastatic, castration-resistant prostate cancer (mCRPC) receive anti-hormonal therapy with androgen receptor signaling inhibitors (ARSi) in order to overcome acquired resistances to first-generation non-steroidal antiandrogens [1].

In clinical practice, the CYP17A1 inhibitor abiraterone acetate and the androgen receptor antagonists enzalutamide, apalutamide and darolutamide are routinely used [1, 2]. A treatment option in advanced mCRPC is radioligand therapy (RLT) targeting the Prostate-Specific Membrane Antigen (PSMA) with [^177^Lu]Lu-PSMA-617 or [^177^Lu]Lu-PSMA-I&T. PSMA is a type II transmembrane glycoprotein that is over-expressed on the surface of prostate cancer cells [3]. The radionuclide ^177^Lu can be coupled to a PSMA ligand, which introduces it into the tumor cell via endocytosis, resulting in localized beta radiation delivery to the tumor DNA. ^177^Lu is a beta emitter (β^−^ emitter) with a maximum energy of 498 keV. It also emits gamma rays (γ-rays), of which 208 keV has the highest emission probability (10.4%), allowing imaging and dosimetric calculations [4–6].

Some studies suggest PSMA expression as measured on PSMA PET can be upregulated in mCRPC patients starting a new ARSi [7, 8]. The question arises whether ARSi could be a potential enhancer medication for PSMA radioligand therapy. Alternatively, it may be the case that this effect is merely a PSMA upregulation phenomenon, devoid of any therapeutic relevance [9].

In the ENZA-p trial, [^177^Lu]Lu-PSMA-617 RLT and enzalutamide therapy were combined in a large-scale prospective study. The results demonstrate that the cohort receiving [^177^Lu]Lu-PSMA-617 RLT concomitantly with enzalutamide exhibited a clear benefit not only in PSA progression-free survival and radiographic progression-free survival, but also in overall survival and key aspects of health-related quality of life, in comparison to patients receiving enzalutamide monotherapy [10, 11].

In this retrospective analysis, we evaluated the absorbed doses in tumor lesions at cycle 1 of [^177^Lu]Lu-PSMA-617 or [^177^Lu]Lu-PSMA-I&T RLT in patients who underwent ARSi treatment concomitantly and compared these with patients who underwent [^177^Lu]Lu-PSMA monotherapy without any ARSi therapy. The aim was to determine if the combination with ARSi therapy enhances the absorbed doses in tumor lesions.

## Methods

### Patients

This retrospective, single-center analysis was conducted on a total of 50 patients diagnosed with mCRPC who underwent their first cycle of [^177^Lu]Lu-PSMA-617 or [^177^Lu]Lu-PSMA-I&T RLT between 2015 and 2021 at the Department of Nuclear Medicine, LMU Munich.

In the Combination Group (*n* = 25), patients received either [^177^Lu]Lu-PSMA-617 or [^177^Lu]Lu-PSMA-I&T radioligand therapy concomitantly with ARSi treatment. Radioligand selection was based on institutional availability. The inclusion criteria for this group mandated that patients had already been receiving an ARSi prior to the initiation of PSMA RLT and continued the ARSi concomitantly with their first cycle of PSMA RLT. A total of 19 patients were treated with [^177^Lu]Lu-PSMA-617, while 6 patients received [^177^Lu]Lu-PSMA-I&T.

In the Control Group (*n* = 25), patients received PSMA RLT as monotherapy. 18 patients were administered [^177^Lu]Lu-PSMA-617, and the remaining 7 patients underwent treatment with [^177^Lu]Lu-PSMA-I&T. The prerequisite for this group was the absence of concomitant ARSi during the first PSMA RLT cycle. However, prior ARSi therapies that had already been discontinued were allowed. The interval between ARSi discontinuation and RLT initiation was not standardized and varied across patients, reflecting the retrospective nature of the study and routine clinical practice.

Across both groups, a broad spectrum of prior treatments was allowed, including local definitive/ablative therapies, systemic regimens and different ARSi exposures. Most patients continued androgen deprivation therapy according to routine clinical practice at our institution. The term “monotherapy” refers to PSMA RLT without concomitant ARSi and does not imply discontinuation of ADT. A detailed overview of prior therapies is provided in Tables 1 and 2.

**Table 1:** Patient characteristics [^177^Lu]Lu-PSMA-617

Parameter	Combination Group (*n* = 19)	Control Group (*n* = 18)
Age at Diagnosis	64 ± 7	63 ± 11
Initial Gleason Score < 8	5	5
Initial Gleason Score ≥ 8	10	8
Initial Gleason Score unknown	4	5
iPSA* in ng/ml	80 ± 170	22 ± 151
PSA prior 1. cycle [^1^⁷⁷Lu]Lu-PSMA-617 in ng/ml	49 ± 246	97 ± 1704
Latency b/w prior PSA and [^1^⁷⁷Lu]Lu-PSMA-617 in days	0 ± 10	0 ± 1
Total tumor volume (TTV) in ml	149 ± 507	570 ± 634
	*p =* 0.14	
Prior definitive/ablative treatments	Combination Group (*n* = 19)	Control Group (*n* = 18)
Radical prostatectomy (RP)	13	12
Focal Therapy	0	2
Definitive Radiotherapy (RT)	1	1
RP + adjuvant RT/SRT	10	9
RT metastases	9	7
Prior Systemic Therapy	Combination Group (*n* = 19)	Control Group (*n* = 18)
ADT only	5	2
ADT + Chemotherapy	4	3
ADT + ^223^Ra	1	2
Chemotherapy + ^223^Ra	0	1
ADT + 1 ARSi	1	1
ADT + 1 ARSi + Chemotherapy	4	2
ADT + 2 ARSi + Chemotherapy	0	1
ADT + 1 ARSi + ^223^Ra	1	0
ADT + 2 ARSi + ^223^Ra	0	1
ADT + 1 ARSi + Chemotherapy + ^223^Ra	2	1
ADT + 2 ARSi + Chemotherapy + ^223^Ra	0	0
1 ARSi + Chemotherapy	0	1
2 ARSi only	0	0
No prior systemic therapy	1	3
Prior ARSi Therapy	Combination Group (*n* = 19)	Control Group (*n* = 18)
Abiraterone	5	3
Enzalutamide	3	2
Abiraterone + Enzalutamide	0	2
No prior ARSi treatment	11	11
ARSi concomitant to [^1^⁷⁷Lu]Lu-PSMA-617	Combination Group (*n* = 19)	
Median latency between new ARSi and 1. cycle in days	272 ± 327	
Age at new ARSi	75 ± 9	
Abiraterone	7	
Enzalutamide	12	

* Available in 6/19; 8/18 patients

**Table 2:** Patient characteristics [^177^Lu]Lu-PSMA-I&T

Parameter	Combination Group (*n* = 6)	Control Group (*n* = 7)
Age at Diagnosis	77 ± 7	68 ± 9
Initial Gleason Score < 8	1	2
Initial Gleason Score ≥ 8	5	5
Initial Gleason Score unknown	0	0
iPSA* in ng/ml	92 ± 209	36 ± 30
PSA prior 1. cycle [^1^⁷⁷Lu]Lu-PSMA-I&T in ng/ml	74 ± 295	161 ± 1031
Latency b/w prior PSA and [^1^⁷⁷Lu]Lu-PSMA-I&T in days	0	0
Total tumor volume (TTV) in ml	258 ± 596	988 ± 523
	*p =* 0.53	
Prior definitive/ablative treatments	Combination Group (*n* = 6)	Control Group (*n* = 7)
Radical prostatectomy (RP)	4	4
Focal Therapy	0	0
Definitive Radiotherapy (RT)	0	2
RP + adjuvant RT/SRT	2	1
RT metastases	2	4
Prior Systemic Therapy	Combination Group (*n* = 6)	Control Group (*n* = 7)
ADT only	1	1
ADT + Chemotherapy	2	0
ADT + ^223^Ra	0	0
Chemotherapy + ^223^Ra	0	0
ADT + 1 ARSi	1	0
ADT + 1 ARSi + Chemotherapy	0	2
ADT + 2 ARSi + Chemotherapy	0	1
ADT + 1 ARSi + ^223^Ra	0	0
ADT + 2 ARSi + ^223^Ra	0	0
ADT + 1 ARSi + Chemotherapy + ^223^Ra	0	1
ADT + 2 ARSi + Chemotherapy + ^223^Ra	0	1
1 ARSi + Chemotherapy	1	0
2 ARSi only	1	0
No prior systemic therapy	0	1
Prior ARSi Therapy	Combination Group (*n* = 6)	Control Group (*n* = 7)
Abiraterone	2	1
Enzalutamide	0	2
Abiraterone + Enzalutamide	1	2
No prior ARSi treatment	3	2
ARSi concomitant to [^1^⁷⁷Lu]Lu-PSMA-I&T	Combination Group (*n* = 6)	
Median latency between new ARSi and 1. cycle in days	271 ± 188	
Age at new ARSi	81 ± 5	
Abiraterone	2	
Enzalutamide	4	

* Available in 6/6; 6/7 patients

All patients provided written informed consent for RLT. The evaluation of these data was approved by local ethics committees.

### Pretherapeutic PET/CT imaging and analysis

Each patient received a pretherapeutic baseline [^68^ Ga]Ga-PSMA-11 PET/CT (*n* = 25) or [^18^F]F-PSMA-1007 PET/CT (*n* = 25) scan to ensure adequate PSMA expression prior to therapy. The use of different PSMA PET tracers was determined by institutional availability.

Furthermore, pretherapeutic PSMA-positive total tumor volume (TTV) was obtained for each patient using Hermes Affinity Viewer (Hermes Medical Solutions, Stockholm, Sweden). For the [^18^F]F-PSMA-1007 PET/CT scans, a uniform threshold of SUV 4.0 was utilized for TTV calculation [12]. For each patient who underwent [^68^ Ga]Ga-PSMA-11 PET/CT, an individual threshold was determined using previously described methods [13].

### [^177^Lu]Lu-PSMA-617 and [^177^Lu]Lu-PSMA-I&T radiopharmaceuticals and treatment protocol

[^177^Lu]Lu-PSMA-617 was obtained by radiolabeling a DOTA-PSMA-617 precursor (ABX GmbH, Radeberg, Germany) with carrierless ^177^LuCl3 (ITG GmbH, Garching, Germany) in accordance with the previously described method [14]. The precursor for [^177^Lu]Lu-PSMA-I&T was provided by Scintomics/ATT GmbH, Fürstenfeldbruck, Germany; radiolabeling was performed in-house.

The radionuclide pharmaceuticals were subsequently injected intravenously over 15–20 min using an infusion pump. Prior to and following the administration of therapy, all patients were hydrated with 2 L of 0.9% NaCl. The treatment was administered in the nuclear medicine department adhering to German radiation protection laws [15]). Patients were given 50 mg of prednisolone and 1 L of 0.9% NaCl intravenously each day until discharge. To mitigate blood flow to the salivary glands and reduce associated side effects, cold packs were applied to the salivary gland regions during and after treatment. Patients were monitored daily through physical examinations and routine blood tests for electrolytes, hematology, liver and kidney function.

### Image acquisition, reconstruction and dosimetry

Quantitative SPECT images were acquired on a Siemens Symbia T2 SPECT/CT system (Siemens Healthineers, Erlangen, Germany) at approximately 24 h, 48 h, and 72 h post administration. At least one CT at 24 h post injection was acquired. Acquisition parameters can be found in our previous publications [16, 17]. The SPECT field-of-view (FOV) was defined based on the pre-therapeutic PSMA PET/CT, aiming to include kidneys and as many PSMA-expressing tumor lesions as possible, as described previously [18], and covered 1 to 3 bed positions.

SPECT images were reconstructed according to the departmental standard protocol [16] using Hermes Hybrid Recon-Oncology 4.0 (Hermes Medical Solutions, Stockholm, Sweden) including the MAP-OSEM (Bayesian weight 0.001, 8s16i). The quantitative reconstruction process involved CT-based attenuation correction, scatter correction, and resolution modeling.

**Fig. 1:**
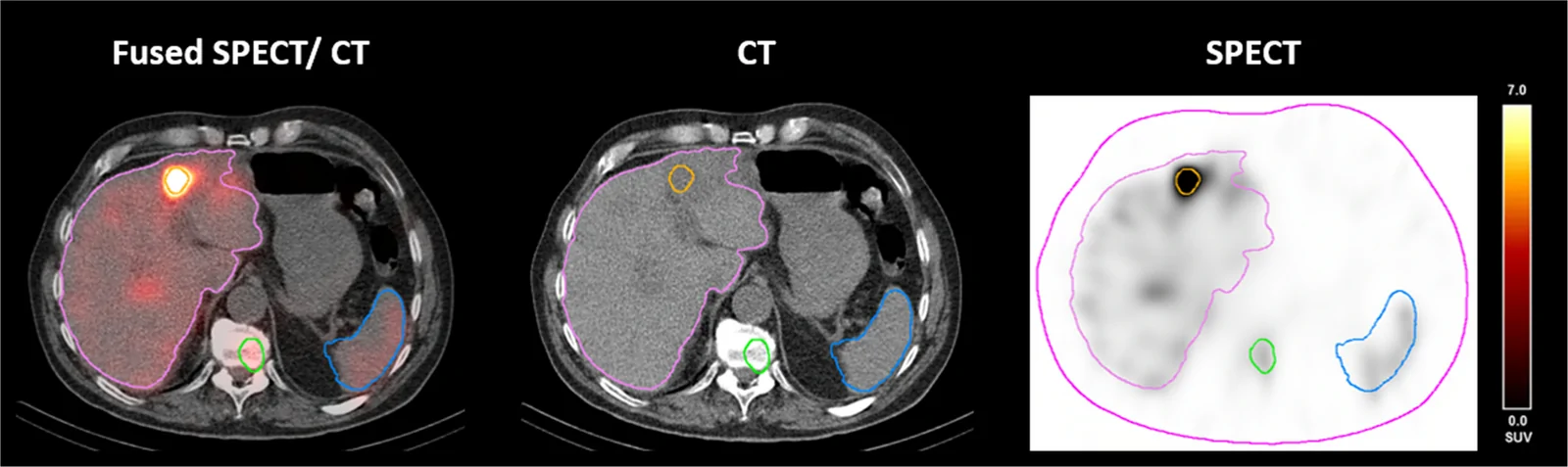
Segmented tumor lesions and organs-at-risk in a patient of the Combination Group receiving [^177^Lu]Lu-PSMA-617

Dosimetry was conducted using MIM SurePlan™ MRT version 7.3.4. (MIM Software, Cleveland, OH, USA). The exemplary organs-at-risk (kidneys, liver, spleen) were manually contoured on the CT (24 h), while all tumor lesions located within the SPECT FOV were segmented on the SPECT at 24 h using the PET Edge + tool (Fig. 1). Time integrated activity curves were created for each segmentation. The absorbed dose calculations are based on a monoexponential curve fit and included local density scaling. The dosimetry results are expressed as median absorbed dose per unit activity (Gy/GBq).

After completion of dosimetry, up to 3 representative lesions from each patient showing the highest median absorbed doses (Gy/GBq) were selected to be included in this study in line with the methodology of other publications [19, 20]. In patients with fewer than 3 tumor lesions within the SPECT FOV, all available lesions were included.

A total of 148 lesions (median 3 lesions per patient) were included for assessing tumor dosimetry, for further specifications see also Tables 3 and 4.

**Table 3:** Dosimetry Results [^177^Lu]Lu-PSMA-617

Parameters	Combination Group (*n* = 19)	Control Group (*n* = 18)
Median injected activity in GBq	6.0 ± 0.5	6.1 ± 1.0
Included tumor lesions	55*	54
Osseous	38	34
Lymphatic	12	18
Visceral	5	2
Median tumor lesion volume in ml	5.04 ± 15.02	6.42 ± 9.38
	*p =* 0.24	
Median tumor absorbed dose in Gy/GBq	2.1 ± 4.0	1.5 ± 1.7
	*p =* 0.24	
Osseous	1.6 ± 3.1	1.8 ± 1.6
	*p =* 1.00	
Lymphatic	3.6 ± 6.0	1.1 ± 1.8
	*p =* 0.00	
Visceral	1.4 ± 1.1	4.4 ± 0.3
	*p =* 0.10	
Median absorbed Doses in organs-at-risk (OAR) in Gy/GBq		
Kidneys	0.28 ± 0.11	0.27 ± 0.13
	*p =* 0.90	
Spleen	0.05 ± 0.05	0.06 ± 0.05
	*p =* 0.75	
Liver	0.08 ± 0.03	0.07 ± 0.04
	*p =* 0.75	

*In 1 patient only 1 visible tumor lesion was observed

**Table 4:** Dosimetry results [^177^Lu]Lu-PSMA-I&T

Parameters	Combination Group (*n* = 6)	Control Group (*n* = 7)
Median injected activity in GBq	8.2 ± 0.9	9.0 ± 1.2
Included tumor lesions	18	21
Osseous	13	12
Lymphatic	5	3
Visceral	0	6
Median tumor lesion volume in ml	3.22 ± 3.95	4.80 ± 8.13
	*p =* 0.03	
Median tumor absorbed dose in Gy/GBq	1.2 ± 1.2	1.2 ± 3.5
	*p =* 0.21	
Osseous	0.6 ± 1.3	0.8 ± 2.6
	*p =* 0.50	
Lymphatic	1.4 ± 0.5	1.2 ± 0.1
	*p =* 0.79	
Visceral	-	3.6 ± 4.8
Median absorbed Doses in organs-at-risk (OAR) in Gy/GBq		
Kidneys	0.21 ± 0.10	0.30 ± 0.08
	*p =* 0.01	
Spleen	0.02 ± 0.02	0.04 ± 0.03
	*p =* 0.20	
Liver	0.02 ± 0.01	0.04 ± 0.01
	*p =* 0.01	

### Statistics

Statistical analyses were performed using GraphPad Prism version 8.4.3 (GraphPad Software, San Diego, CA, USA). Descriptive statistics were summarized as medians for the dosimetry results. A Mann–Whitney U test was performed to compare the dosimetry outcomes between Combination Group and Control Group, with an alpha level of 0.05 for statistical significance.

## Results

### Patient characteristics

Overall, the median age at diagnosis was 64 ± 9 years. Specifically, the median age at diagnosis for patients in the Combination Group was 66 ± 9 years, while the median age for those in the Control Group was 64 ± 10 years.

The overall median PSA level prior to the first cycle of RLT was 63 ± 1121 ng/ml. In particular, the median PSA was observed to be 49 ± 253 ng/ml in the Combination Group and 138 ± 1525 ng/ml in the Control Group. For further specifications, see Tables 1 and 2.

The pretherapeutic PSMA-positive total tumor volume (TTV) in both the PSMA-617 and PSMA-I&T subgroups showed no significant difference ([^177^Lu]Lu-PSMA-617: 149 ml ± 507 ml vs 570 ml ± 634 ml, *p =* 0.14; [^177^Lu]Lu-PSMA-I&T: 258 ml ± 596 ml vs 988 ml ± 523 ml, *p =* 0.53).

In the Control Group, 12/25 patients (48%) received prior ARSi therapy, which had been discontinued before initiation of PSMA RLT. The interval between ARSi discontinuation and the first RLT cycle varied widely across patients, reflecting the absence of a standardized washout interval and the retrospective nature of the study.

The Combination Group, which received ARSi treatment concomitant to the initial cycle of [^177^Lu]Lu-PSMA-617 or [^177^Lu]Lu-PSMA-I&T, exhibited a median latency between the initiation of therapy with ARSi and the first cycle of 272 ± 298 days. 9/25 patients (36%) in the Combination Group received abiraterone and 16/25 (64%) received enzalutamide. See Tables 1 and 2 for additional specifications.

### Absorbed doses in control group versus combination group

In our analysis, we evaluated dosimetry results for patients treated with [^177^Lu]Lu-PSMA-617 and [^177^Lu]Lu-PSMA-I&T separately, as these two radioligands exhibit differences in their pharmacokinetics, biodistribution, and affinity for PSMA.

For [^177^Lu]Lu-PSMA-617, the median injected activity in the Combination Group was 6.0 ± 0.5 GBq, whereas the Control Group exhibited a median injected activity of 6.1 ± 1.0 GBq (Table 3). Patients treated with [^177^Lu]Lu-PSMA-I&T had a median injected activity in the Combination Group of 8.2 ± 0.9 GBq compared to 9.0 ± 1.2 GBq for the Control Group (Table 4).

The median tumor absorbed dose was found to be comparable between the Combination Group and the Control Group, with no statistically significant difference observed for both [^177^Lu]Lu-PSMA-617 (2.1 ± 4.0 Gy/GBq vs 1.5 ± 1.7 Gy/GBq, *p =* 0.24) and [^177^Lu]Lu-PSMA-I&T (1.2 ± 1.2 Gy/GBq vs 1.2 ± 3.5 Gy/GBq, *p =* 0.21). For further specifications see Tables 3, 4 and Supplementary Figs. 1, 2.

### Absorbed doses in organs-at-risk

For dosimetry of organs-at-risk (OAR), no significant difference was identified in the median absorbed dose between Combination Group and Control Group for [^177^Lu]Lu-PSMA-617 (Kidneys: 0.28 ± 0.11 Gy/GBq vs 0.27 ± 0.13 Gy/GBq, *p =* 0.90; Spleen: 0.05 ± 0.05 Gy/GBq vs 0.06 ± 0.05 Gy/GBq, *p =* 0.75; Liver: 0.08 ± 0.03 Gy/GBq vs 0.07 ± 0.04 Gy/GBq, *p =* 0.75).

For [^177^Lu]Lu-PSMA-I&T, a statistically significant difference was identified between the Combination Group and the Control Group with regard to the median absorbed dose in kidneys and liver, whereas no significant difference was observed in the spleen (Kidneys: 0.21 ± 0.10 Gy/GBq vs 0.30 ± 0.08 Gy/GBq, *p =* 0.01; Spleen: 0.02 ± 0.02 Gy/GBq vs 0.04 ± 0.03 Gy/GBq, *p =* 0.20; Liver: 0.02 ± 0.01 Gy/GBq vs 0.04 ± 0.01 Gy/GBq, *p =* 0.01). For further details please see Tables 3, 4 and Supplementary Figs. 3, 4.

## Discussion

[^177^Lu]Lu-PSMA-617 and [^177^Lu]Lu-PSMA-I&T radioligand therapy have become a promising treatment option for patients with metastatic castration-resistant prostate cancer (mCRPC). The VISION trial (NCT03511664) proved the effectiveness of [^177^Lu]Lu-PSMA-617 in enhancing radiographic progression-free survival as well as overall survival in patients diagnosed with mCRPC [21]. Parallel to the development of PSMA-targeted therapies, androgen receptor signaling inhibitors (ARSi), including enzalutamide and abiraterone, were introduced as a relevant therapeutic approach in mCRPC management.

Recent research highlights the potential of ARSi to upregulate PSMA expression in mCRPC patients. This prompts the question of whether the combination of ARSi and [^177^Lu]Lu-PSMA-617 or [^177^Lu]Lu-PSMA-I&T RLT could enhance the treatment’s efficacy by increasing uptake of the therapeutic substance and therefore an enhanced radiation delivery to tumor lesions [7–9, 22]. The ENZA-p trial (NCT04419402) demonstrated a clear therapeutic benefit of combining enzalutamide with [^177^Lu]Lu-PSMA-617, compared to enzalutamide monotherapy, leading to the hypothesis of a potential synergistic effect between ARSi and PSMA-targeted therapy [10]. This has led to the hypothesis of a potential synergistic effect between ARSi and PSMA therapy. It should be noted, however, that ENZA-P addressed a different clinical question by comparing enzalutamide plus PSMA RLT with enzalutamide monotherapy and therefore does not directly address the comparison of PSMA RLT with versus without concomitant ARSi in our study. One proposed mechanism is that the potential upregulation of PSMA expression, caused by the androgen receptor signaling inhibition of enzalutamide, could make cancer cells more susceptible to PSMA-targeted therapies leading to an increased uptake of the radiopharmaceutical in prostate cancer cells.

However, studies suggest that such a PSMA upregulation is most pronounced within a short time window after ARSi initiation, whereas in our study ARSi treatment in the Combination Group had been ongoing for several months prior to the first cycle of PSMA RLT.

In our analysis, the addition of ARSi did not have a significant impact on tumor absorbed dose during the first cycle of [^1^⁷⁷Lu]Lu-PSMA-617 or [^1^⁷⁷Lu]Lu-PSMA-I&T therapy.

These results may provide an initial indication that the observed therapeutic effect might be purely additive, with both therapies acting independently, rather than synergistic.

Importantly, this interpretation is exclusively based on lesion dosimetry and does not imply improved clinical response or efficacy, which were not assessed in this analysis.

Regarding lesion size and potential partial volume effects, tumor lesions were, on average, larger in the [^1^⁷⁷Lu]Lu-PSMA-I&T Control Group than in the Combination Group. This difference would be expected to cause higher lesion recovery in the Control Group. However, the finding that ARSi does not lead to higher lesion absorbed doses would still be valid. In the [^1^⁷⁷Lu]Lu-PSMA-617 cohort, lesion volumes were comparable between the Combination Group and the Control Group.

Moreover, this interpretation must be viewed considering the heterogeneous timing of ARSi exposure, which may have attenuated potential short-term ARSi-related effects at the time of dosimetry.

Comparably, a multicenter retrospective study found that only 25% of patients with metastatic castration-resistant prostate cancer show ≥ 30% PSMA-TV increases within 30 days of ARSI initiation [23]. This increased overexpression may be more common in less heavily pretreated patients, and it seems to be intense only in a minority of patients. A secondary PET imaging analysis of the ENZA-P trial supports this distinction: in the combination group the baseline PSMA PET SUV (reflecting the PSMA Target expression level) lost prognostic power, suggesting that a parallel targeting (AR plus PSMA [i.e., additive effect]) rather than synergistic flare-driven uptake explains the improved outcomes [24].

Similar to the tumor dosimetry outcomes, only minor differences were observed in the absorbed dose of organs-at-risk (OAR). This highlights the absence of significant adverse effects for OAR associated with the combination of both therapies. A notable aspect is the significant difference in the median absorbed dose of kidneys and liver in patients receiving [^177^Lu]Lu-PSMA-I&T. This may be attributed to the relatively small size of the group (*n* = 13).

These key findings indicate that the addition of ARSi did not have a measurable impact on the tumor absorbed dose after the first cycle of [^177^Lu]Lu-PSMA-617 or [^177^Lu]Lu-PSMA-I&T therapy in this pilot cohort. Our results provide useful context in the ongoing discourse regarding the combination of ARSi with PSMA-targeted therapies.

It should be noted that our study has several limitations: First, the retrospective study design introduces potential biases, which may affect the validity of the results. Secondly, patients of the Combination Group received ARSi pretreatment for a median of 272 ± 298 days: As recent literature describes that PSMA upregulation may occur after a short-term ARSi treatment lasting < 30 days [8], various pretreatment intervals between ARSi and onset of PSMA therapy should be evaluated in further studies. In addition, some patients in the Control Group had received ARSi therapy prior to PSMA RLT with variable discontinuation intervals, which may have attenuated potential group differences.

Furthermore, our analysis is only focused on the first cycle of [^177^Lu]Lu-PSMA-617 or [^177^Lu]Lu-PSMA-I&T therapy and is including patients with different ARSi agents and different (ARSi-) pretreatments. This approach was chosen to ensure comparability in this retrospective pilot study, as dosimetry in later cycles may be influenced by therapy induced changes. Here, further analyses are needed that are also including the assessment of more cycles of PSMA therapy under concomitant ARSi treatment. Furthermore, the small sample size limits statistical power and may reduce the generalizability of our results. It also should be noted that the dosimetry values presented in this study were not adjusted for partial volume effects (PVE), which may lead to an underestimation of absorbed doses in particular for small lesions. To further address this, an additional subgroup analysis restricted to tumor lesions ≥ 3 ml was performed, which revealed no significant differences in tumor absorbed dose between the Combination and Control Groups, confirming our main findings. Additionally, further studies have to respect the absorbed dose of the whole-body tumor volume and should not only be focused on exemplary lesions as we performed in this study.

Our pilot study suggests that the therapeutic advantages of combining ARSi with PSMA-based therapy may be influenced by additional factors, including treatment timing, disease progression, and the interval between the initiation of ARSi and the initial cycle of PSMA therapy.

Given the rapidly evolving treatment landscape of mCRPC, it is important to consider the potential clinical implications of our findings. In recent years, ARSi are being increasingly used earlier in the disease course, particularly in patients with metastatic hormone-sensitive prostate cancer (mHSPC). This raises the relevant clinical question of whether an ARSi rechallenge, at the time of mCRPC progression, could represent a valuable therapeutic option when combined with PSMA RLT. Our results suggest that such a combination may provide an additive rather than synergistic effect in terms of tumor absorbed dose. However, considering the possible benefit of ARSi rechallenge in selected mCRPC patients, future prospective studies should systematically explore optimal treatment sequencing and timing of ARSi in combination with PSMA-targeted therapy and should implement dosimetry in the study design.

Further investigation, including prospective, multi-center studies with larger patient cohorts and standardized treatment protocols, is required to fully understand the potential clinical advantages of ARSi and PSMA RLT.

## Conclusion

This retrospective study provides the first dosimetric analysis of mCRPC patients treated with [^177^Lu]Lu-PSMA-617 or [^177^Lu]Lu-PSMA-I&T therapy in combination with androgen receptor signaling inhibitors (ARSi). It could be shown that the concomitant administration of ARSi does not result in a statistically significant increase in tumor absorbed doses after one cycle PSMA therapy. Further investigation, including the implementation of larger prospective trials, is required to fully understand the potential effects of combining PSMA therapy with ARSi.

## Supplementary Information

Below is the link to the electronic supplementary material.Supplementary file1 (DOCX 31 KB)

## Data Availability

The herein presented data is available in case of a reasonable request from the corresponding author.

## References

[1] Tilki D, van den Bergh RCN, Briers E, Van den Broeck T, Brunckhorst O, Darraugh J, et al. EAU-EANM-ESTRO-ESUR-ISUP-SIOG guidelines on prostate cancer. Part II-2024 update: treatment of relapsing and metastatic prostate cancer. Eur Urol. 2024;86:164–82. 10.1016/j.eururo.2024.04.010. 38688773

[2] Jacob A, Raj R, Allison DB, Myint ZW. Androgen receptor signaling in prostate cancer and therapeutic strategies. Cancers (Basel). 2021. 10.3390/cancers13215417. 34771580 PMC8582395

[3] Parsi M, Desai MH, Desai D, Singhal S, Khandwala PM, Potdar RR. PSMA: a game changer in the diagnosis and treatment of advanced prostate cancer. Med Oncol. 2021;38:89. 10.1007/s12032-021-01537-3. 34181109

[4] Unterrainer LM, Calais J, Bander NH. Prostate-specific membrane antigen: gateway to management of advanced prostate cancer. Annu Rev Med. 2024;75:49–66. 10.1146/annurev-med-081522-031439. 38285513

[5] Tagawa ST, Milowsky MI, Morris M, Vallabhajosula S, Christos P, Akhtar NH, et al. Phase II study of Lutetium-177-labeled anti-prostate-specific membrane antigen monoclonal antibody J591 for metastatic castration-resistant prostate cancer. Clin Cancer Res. 2013;19:5182–91. 10.1158/1078-0432.CCR-13-0231. 23714732 PMC3778101

[6] Ljungberg M, Celler A, Konijnenberg MW, Eckerman KF, Dewaraja YK, Sjogreen-Gleisner K. MIRD pamphlet No. 26: joint EANM/MIRD guidelines for quantitative 177Lu SPECT applied for dosimetry of radiopharmaceutical therapy. J Nucl Med. 2016;57:151–62. 10.2967/jnumed.115.159012. 26471692

[7] Rosar F, Bader H, Bartholoma M, Maus S, Burgard C, Linxweiler J, et al. Addition of standard Enzalutamide medication shows synergistic effects on response to [(177)Lu]Lu-PSMA-617 radioligand therapy in mCRPC patients with imminent treatment failure-preliminary evidence of pilot experience. Cancers (Basel). 2022. 10.3390/cancers14112691. 35681671 PMC9179420

[8] Rosar F, Neher R, Burgard C, Linxweiler J, Schreckenberger M, Hoffmann MA, et al. Upregulation of PSMA expression by Enzalutamide in patients with advanced mCRPC. Cancers (Basel). 2022. 10.3390/cancers14071696. 35406467 PMC8997007

[9] Sonni I, Gafita A, Unterrainer LM, Alano RM, Lira S, Shen J, et al. Effects of novel androgen receptor signaling inhibitors on PSMA PET signal intensity in patients with castrate-resistant prostate cancer: a prospective exploratory serial imaging study. EJNMMI Res. 2023;13:95. 10.1186/s13550-023-01048-4. 37902861 PMC10616012

[10] Emmett L, Subramaniam S, Crumbaker M, Joshua AM, Sandhu S, Nguyen A, et al. Overall survival and quality of life with [(177)Lu]Lu-PSMA-617 plus enzalutamide versus enzalutamide alone in metastatic castration-resistant prostate cancer (ENZA-p): secondary outcomes from a multicentre, open-label, randomised, phase 2 trial. Lancet Oncol. 2025;26:291–9. 10.1016/S1470-2045(25)00009-9. 39956124

[11] Emmett L, Subramaniam S, Crumbaker M, Nguyen A, Joshua AM, Weickhardt A, et al. [(177)Lu]Lu-PSMA-617 plus enzalutamide in patients with metastatic castration-resistant prostate cancer (ENZA-p): an open-label, multicentre, randomised, phase 2 trial. Lancet Oncol. 2024;25:563–71. 10.1016/S1470-2045(24)00135-9. 38621400

[12] Unterrainer LM, Beyer L, Zacherl MJ, Gildehaus FJ, Todica A, Kunte SC, et al. Total tumor volume on (18)F-PSMA-1007 PET as additional imaging biomarker in mCRPC patients undergoing PSMA-targeted alpha therapy with (225)Ac-PSMA-I&T. Biomedicines. 2022. 10.3390/biomedicines10050946. 35625683 PMC9138410

[13] Seifert R, Herrmann K, Kleesiek J, Schäfers M, Shah V, Xu Z, et al. Semiautomatically quantified tumor volume using (68)Ga-PSMA-11 PET as a biomarker for survival in patients with advanced prostate cancer. J Nucl Med. 2020;61:1786–92. 10.2967/jnumed.120.242057. 32332147

[14] Fendler WP, Reinhardt S, Ilhan H, Delker A, Boning G, Gildehaus FJ, et al. Preliminary experience with dosimetry, response and patient reported outcome after 177Lu-PSMA-617 therapy for metastatic castration-resistant prostate cancer. Oncotarget. 2017;8:3581–90. 10.18632/oncotarget.12240. 27683041 PMC5356905

[15] German Radiation Protection Ordinance (Strahlenschutzverordnung – StrlSchV). Ordinance on protection against the harmful effects of ionising radiation. Bonn, Germany: Federal Law Gazette (Bundesgesetzblatt), Part I; 2018. p. 2034–6

[16] Resch S, Takayama Fouladgar S, Zacherl M, Sheikh GT, Liubchenko G, Rumiantcev M, et al. Investigation of image-based lesion and kidney dosimetry protocols for (177)Lu-PSMA-I&T therapy with and without a late SPECT/CT acquisition. EJNMMI Phys. 2023;10:11. 10.1186/s40658-023-00529-8. 36757516 PMC9911578

[17] Delker A, Fendler WP, Kratochwil C, Brunegraf A, Gosewisch A, Gildehaus FJ, et al. Dosimetry for (177)Lu-DKFZ-PSMA-617: a new radiopharmaceutical for the treatment of metastatic prostate cancer. Eur J Nucl Med Mol Imaging. 2016;43:42–51. 10.1007/s00259-015-3174-7. 26318602

[18] Delker A, Schleske M, Liubchenko G, Berg I, Zacherl MJ, Brendel M, et al. Biodistribution and dosimetry for combined [(177)Lu]Lu-PSMA-I&T/[(225)Ac]Ac-PSMA-I&T therapy using multi-isotope quantitative SPECT imaging. Eur J Nucl Med Mol Imaging. 2023;50:1280–90. 10.1007/s00259-022-06092-1. 36629878 PMC10027798

[19] Völter F, Mittlmeier L, Gosewisch A, Brosch-Lenz J, Gildehaus FJ, Zacherl MJ, et al. Correlation of an index-lesion-based SPECT dosimetry method with mean tumor dose and clinical outcome after (177)Lu-PSMA-617 radioligand therapy. Diagnostics Basel. 2021. 10.3390/diagnostics11030428. 33802417 PMC7999994

[20] Okamoto S, Thieme A, Allmann J, D’Alessandria C, Maurer T, Retz M, et al. Radiation dosimetry for (177)Lu-PSMA I&T in metastatic castration-resistant prostate cancer: absorbed dose in normal organs and tumor lesions. J Nucl Med. 2017;58:445–50. 10.2967/jnumed.116.178483. 27660138

[21] Sartor O, de Bono J, Chi KN, Fizazi K, Herrmann K, Rahbar K, et al. Lutetium-177-PSMA-617 for metastatic castration-resistant prostate cancer. N Engl J Med. 2021;385:1091–103. 10.1056/NEJMoa2107322. 34161051 PMC8446332

[22] Staniszewska M, Fragoso Costa P, Eiber M, Klose JM, Wosniack J, Reis H, et al. Enzalutamide enhances PSMA expression of PSMA-low prostate cancer. Int J Mol Sci. 2021. 10.3390/ijms22147431. 34299051 PMC8304389

[23] Unterrainer LM, Farolfi A, Grogan T, Hotta M, Djaileb L, Gafita A, et al. Early changes of PSMA PET expression after initiation of androgen receptor signaling inhibitors in CRPC: an international multicenter retrospective study. Eur J Nucl Med Mol Imaging. 2025;52:3700–8. 10.1007/s00259-025-07178-2. 40137974

[24] Emmett L, Papa N, Subramaniam S, Crumbaker M, Nguyen A, Joshua AM, et al. Prognostic and predictive value of baseline PSMA-PET total tumour volume and SUVmean in metastatic castration-resistant prostate cancer in ENZA-p (ANZUP1901): a substudy from a multicentre, open-label, randomised, phase 2 trial. Lancet Oncol. 2025. 10.1016/S1470-2045(25)00339-0. 40752515

