# Supplementary material for: Effects of androgen receptor signaling inhibitors on absorbed doses in mCRPC patients undergoing [177Lu]Lu-PSMA-617 or [177Lu]Lu-PSMA-I&T therapy: dosimetry results
Source: Eur J Nucl Med Mol Imaging. 2026 Jun 11;53(11):6442–51. doi: 10.1007/s00259-026-07967-3 (PMC13526123; doi:10.1007/s00259-026-07967-3)
Supplement: Supplementary file 1 [file 259_2026_7967_MOESM1_ESM.docx]

**SUPPLEMENTARY MATERIAL**

*Effects of androgen receptor signaling inhibitors on absorbed doses in mCRPC patients undergoing [^177^Lu]Lu-PSMA-617 or [^177^Lu]Lu-PSMA-I&T therapy:
dosimetry results*

**Supp. 1:** Median tumor lesion absorbed dose of [^177^Lu]Lu-PSMA-617

**Supp. 2:** Median tumor lesion absorbed dose of [^177^Lu]Lu-PSMA-I&T

**Supp. 3:** Median absorbed dose in OAR of [^177^Lu]Lu-PSMA-617

**Supp. 4:** Median absorbed dose in OAR of [^177^Lu]Lu-PSMA-I&T
